# Single-stage replantation in crush hand amputation: Decision-making beyond injury mechanism

**DOI:** 10.1016/j.jpra.2026.05.004

**Published:** 2026-05-09

**Authors:** Thach Ngoc Nguyen, Thi Cao

**Affiliations:** a Hospital for Traumatology and Orthopaedics, Ho Chi Minh City. 929 Tran Hung Dao Street, Ward Cho Quan, Ho Chi Minh City, Vietnam; b University of Medicine and Pharmacy at Ho Chi Minh City, 217 Hong Bang Street, Ward Cho Lon, Ho Chi Minh City, Vietnam

**Keywords:** Replantation, Crush injuries, Decision making, Microsurgery, Treatment outcome

## Abstract

Hand-level amputation following crush injury presents a major reconstructive challenge, and the choice between single-stage and staged replantation remains controversial. Decision-making is often influenced by the injury mechanism; however, the role of reconstructability is less clearly defined.

We report a case of complete traumatic hand amputation due to crush injury treated with single-stage replantation and long-term follow-up. The amputated segment was reconstructable, allowing direct repair of vessels, nerves, and tendons without grafting. Stable skeletal fixation and tension-free anastomosis were achieved within a reasonable operative time.

The replanted hand survived without vascular complications. At 9-year follow-up, the patient achieved functional use of the hand in daily activities and returned to manual work despite residual limitation in range of motion.

This case highlights that crush injury alone should not preclude a single-stage approach. A simplified decision-making framework is proposed, emphasizing reconstructability and the feasibility of tension-free repair as key determinants. Functional outcomes should be interpreted in terms of practical use rather than maximal joint motion.

## Introduction

Hand-level amputation following crush injury presents a major reconstructive challenge due to extensive soft-tissue damage and difficulty in assessing tissue viability. A staged approach is often considered to reduce operative risk. However, criteria for selecting single-stage replantation in crush injuries remain unclear. We present a case highlighting decision-making factors that may support this approach.

### Decision-making and technical considerations

Crush injury alone should not be regarded as an absolute contraindication to single-stage replantation. Rather, the decision should be guided by reconstructability and the ability to achieve stable fixation and secure vascular reconstruction within a reasonable operative time.

In this case, the amputated segment remained reconstructable despite the crush mechanism, with identifiable vessels, nerves, and tendons suitable for direct repair without grafting. Minimal bone shortening (approximately 2 mm) and limited arterial trimming (approximately 1 mm) enabled tension-free anastomosis. Stable skeletal fixation was achieved using buried Kirschner wires without transarticular pinning, preserving joint mobility and facilitating early rehabilitation.

Careful debridement removed non-viable tissue while preserving essential structures, maintaining adequate length and tissue quality. These factors collectively allowed restoration of vascular inflow and outflow, neural continuity, and tendon function in a single stage within approximately 5 h.

Importantly, the feasibility of a single-stage approach depends not only on structural integrity but also on the ability to complete reconstruction within a physiologically acceptable operative duration. While no strict threshold exists, prolonged operative time may increase systemic risk and should be considered in strategy selection.

A single-stage strategy may not be appropriate in all crush injuries. Extensive soft-tissue destruction, severe contamination, the need for complex vascular or soft-tissue grafting, or anticipated prolonged operative time may favor a staged approach. Therefore, intraoperative assessment of reconstructability should guide decision-making rather than injury mechanism alone.

### Illustrative case

**Fig. 1:**
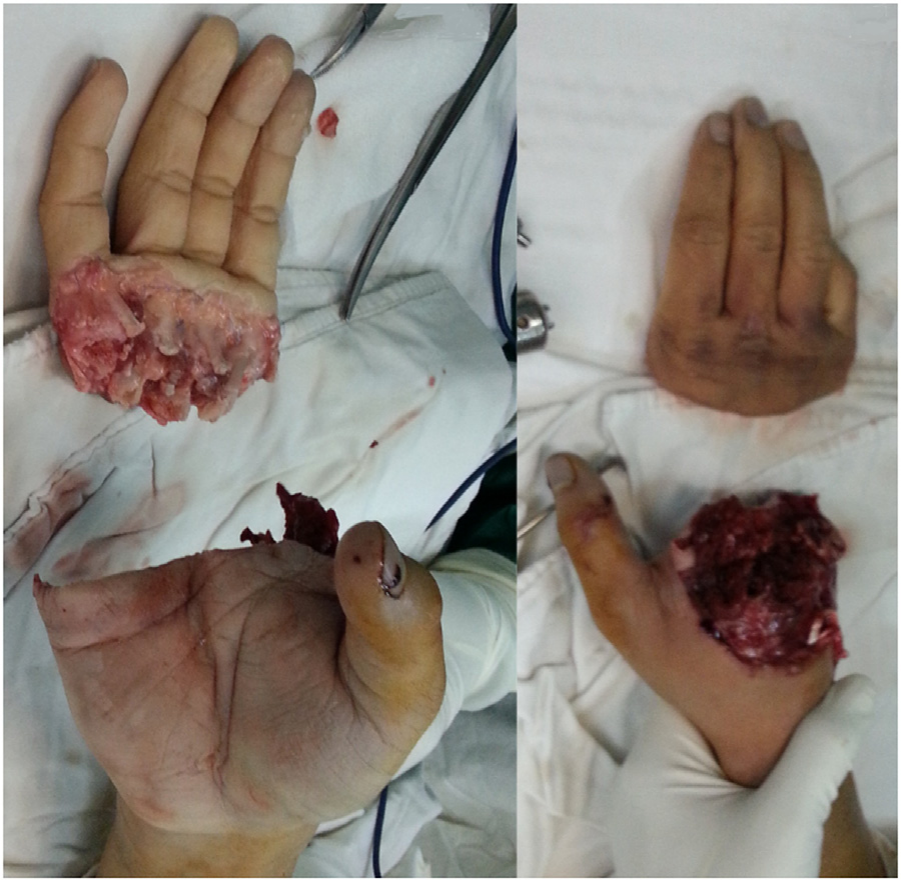
Preoperative appearance of the amputated hand. Clinical photograph showing a complete traumatic amputation of the right hand caused by a crush injury at the metacarpal neck level of digits II–V, with extensive damage to bone, tendons, vessels, and nerves.

A right-hand–dominant male sustained a complete traumatic amputation of the right hand at the metacarpal neck level of digits II–V following a work-related crush injury (Fig. 1).

Single-stage replantation was performed with an operative time of approximately 5 h. After debridement, minimal bone shortening and arterial trimming allowed tension-free reconstruction. Direct end-to-end vascular and nerve repairs were achieved without grafting. Skeletal fixation was performed using buried Kirschner wires without joint transfixation, followed by tendon repair.

The replanted hand survived without vascular complications. Rehabilitation was initiated, and only one secondary procedure—hardware removal with extensor tenolysis—was required at 12 months.

### Functional outcome

At 9-year follow-up, the hand remained viable, with no chronic pain or cold intolerance. Sensory recovery was moderate, with static two-point discrimination of 8–10 mm.

**Fig. 2:**
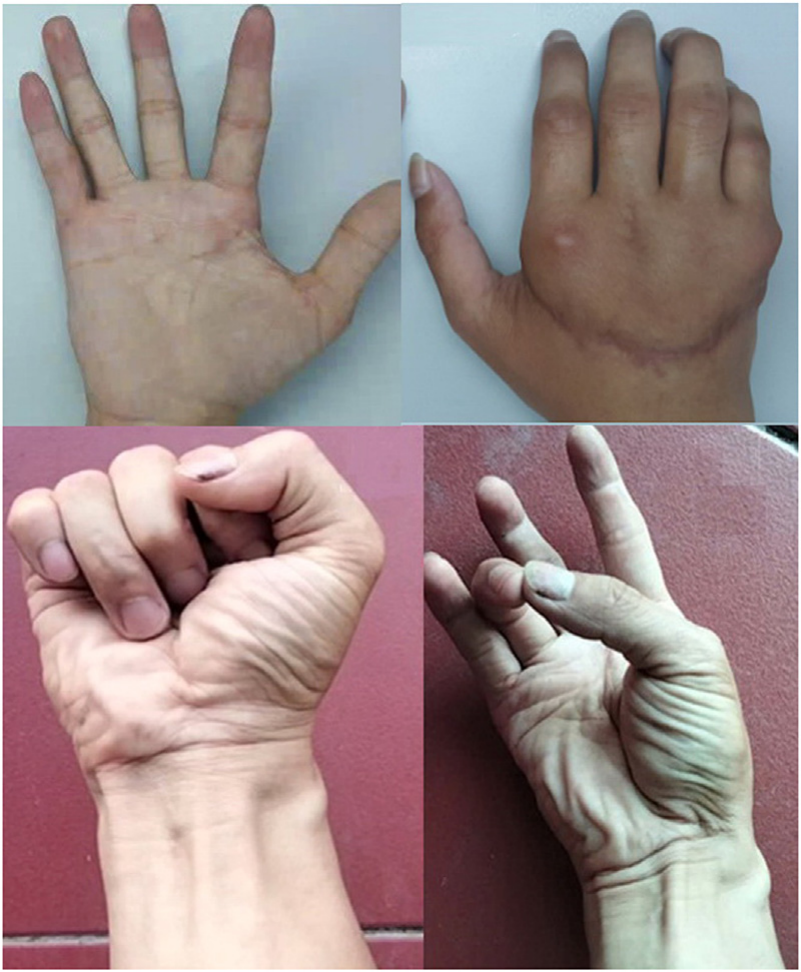
Long-term functional outcome of the replanted hand. Clinical photographs at 9-year follow-up demonstrating palmar and dorsal views of the replanted hand with preserved finger motion, thumb opposition.

Although range of motion remained limited, particularly in extension, the patient achieved functional grasp, pinch, and release. He returned to manual work using the replanted hand as the dominant hand and reported satisfaction (Fig. 2). Functional use may be achieved even without full range of motion.^1^

## Discussion

The choice between single-stage and staged replantation in crush injuries remains debated. While staged approaches may be advantageous in cases with uncertain tissue viability or high complexity, a single-stage strategy can be effective when key reconstructive conditions are met. Functional outcomes after replantation are influenced by multiple factors beyond the mechanism of injury.^2^

Reconstructability appears to be a more relevant determinant than injury mechanism. The ability to achieve secure vascular repair without grafting and stable skeletal fixation is critical, whereas extensive soft-tissue loss or segmental vascular defects may favor staged procedures.^3^

Based on this case, a simplified decision-making pathway may be proposed. Initial assessment should focus on reconstructability, including identifiable vessels, nerves, and tendons suitable for direct repair. If vascular anastomosis can be achieved without complex grafting and stable fixation is feasible, a single-stage approach may be considered. Conversely, when there is extensive tissue destruction, segmental vascular loss, severe contamination, or prolonged operative time is anticipated, a staged strategy may be more appropriate.

This structured approach emphasizes that decision-making should be guided by reconstructive feasibility rather than injury mechanism alone. Importantly, this perspective reframes crush injury as a relative rather than absolute determinant, shifting surgical decision-making away from injury mechanism toward reconstructive feasibility.

Secondary procedures are common and may influence treatment burden.^4^ In this case, only one secondary intervention was required, suggesting that appropriate selection may reduce the need for repeated procedures. Functional outcomes should be interpreted in terms of practical use rather than isolated joint motion.^5^

## Conclusion

Single-stage replantation can be considered in selected crush hand amputations when reconstructability allows tension-free repair within a reasonable operative time. Decision-making should be guided by surgical feasibility and functional goals rather than injury mechanism alone.

## Funding

This study was funded entirely by its author.

## Ethics and consent

Formal ethics committee approval was not required according to institutional policy for this single-case report. Written informed consent was obtained from the patient for publication of clinical information and images, and all identifying information was anonymized.

## Declaration of competing of interest

All authors have no conflict of interest and any authorship issue had been solved before the manuscript was submitted.
